# Reduction of neonatal intensive care unit (NICU) parental perceptions of child vulnerability and risk of vulnerable child syndrome utilizing cognitive behavioral therapy: randomized controlled trial

**DOI:** 10.1038/s41390-025-04094-x

**Published:** 2025-05-15

**Authors:** Margaret K. Hoge, Elizabeth Heyne, Steven Brown, Roy Heyne, Richard J. Shaw, Lina Chalak

**Affiliations:** 1 Department of Pediatrics, University of Texas Southwestern Medical Center, Dallas, TX USA; 2 Department of Health Systems Research, Parkland Health, Dallas, TX USA; 3 Department of Psychiatry and Behavioral Sciences, Stanford University School of Medicine, Palo Alto, CA USA

## Abstract

**Background:**

Neonatal intensive care unit (NICU) parental emotional trauma can distort parental perceptions of child vulnerability (PPCV), resulting in adverse child developmental outcomes, known as Vulnerable Child Syndrome (VCS). We hypothesize utilizing a novel trauma-informed cognitive behavioral therapy (CBT) intervention will reduce PPCV in premature NICU infants’ parents.

**Methods:**

English and Spanish speaking parents of preterm infants (<31 weeks gestational age) were randomized from April 2019 to March 2020 to receive either a 5-session trauma-informed CBT intervention created for this study educating parents on PPCV concepts, or to a control group receiving standard of care. Principal outcome measure was PPCV change measured by the Vulnerable Baby Scale (VBS) scores from enrollment (33 weeks post menstrual age) to study end (6 months chronological age).

**Results:**

8 control and 12 intervention families completed the study (*n* = 42 randomized) due to COVID-19 mandatory research pause. CBT intervention group had a median VBS decrease of 6 points vs. 0 point in controls (*P* = 0.07). Post-hoc Bayesian analysis of VBS PPCV reduction (utilized due to limited *n*) favored CBT to control by 95%.

**Conclusion:**

This is the first parental trauma-informed CBT intervention to demonstrate a PPCV decrease and lower risk of development of VCS in a high-risk NICU population.

**Impact:**

A brief intervention shows promise in fostering improved parenting perceptions, behaviors, and outcomes. NICU parental trauma negatively impacts parental perceptions of child vulnerability and their parenting styles resulting in poor child developmental outcomes, summarized as Vulnerable Child Syndrome (VCS). Currently, there is no effective treatment standard of care to address this important clinical issue. This manuscript contributes to our understanding of the following:

- Trauma-informed cognitive behavioral therapy lowers parents’ perceptions of vulnerability in an at-risk NICU population.
- This is the first published intervention to demonstrate efficacy in reducing NICU parental perceptions of child vulnerability, a key contributor to VCS.
- Implementation of this intervention with NICU families has the potential to reduce the risk of VCS and improve parent-child outcomes and child developmental outcomes.

**Clinical Trial Registration:**

ClinicalTrials.gov identifier: NCT03906435.

## Introduction

It is well-established that the Neonatal Intensive Care Unit (NICU) admission experience invokes parental trauma and is associated with higher rates of parental depression, anxiety and posttraumatic stress disorder (PTSD) compared with parents of term healthy infants.^1–6^ NICU parents have two to five times the risk of clinically significant mental health needs compared to the general population with about 50% of NICU parents meeting clinical criteria for depression and anxiety, and about 40–80% developing PTSD.^5,7–9^

Parent mental health ramifications encountered during a child’s NICU admission often can inflate and distort parental perceptions of their child’s vulnerability (PPCV). Up to 64% of NICU parents have heightened PPCV.^10^ PPCV results in an overprotective parenting pattern, which can affect child developmental outcomes, defined as Vulnerable Child Syndrome (VCS).^11^ VCS is associated with poor child outcomes with respect to the child’s neurodevelopment, behaviors, and medical and psychological health.^11^

Originally, it was suggested that VCS could be prevented^11^; however, there have been no studies demonstrating this thus far. A NICU VCS theoretical model showed that maternal responses to trauma, depression, and anxiety were most influential in the development of increased PPCV and VCS.^12–16^ Trauma-focused cognitive behavioral therapy (CBT) interventions are effective for trauma, depression, and anxiety in a manualized format for a NICU population.^5^ One study examined CBT as an intervention for PPCV early in a NICU admission, however, no significant differences were found potentially due to content of the CBT and timing of the intervention.^13^

We wanted to test whether a novel trauma-focused CBT intervention educating parents on NICU trauma’s effects on PPCV surrounding NICU discharge would reduce PPCV and thereby reduce VCS risk in a NICU population. We hypothesize utilizing our novel trauma-informed cognitive behavioral therapy (CBT) intervention surrounding NICU discharge will reduce PPCV in premature NICU infants’ parents.

## Methods

### Participants

Subjects were English and Spanish speaking mothers of preterm infants (<31 weeks gestational age). Participants were recruited in Dallas, TX at Parkland Health and Hospital Systems NICU starting April 2019 and followed through April 2020 at the Children’s Health NICU follow-up clinic. Inclusion criteria: 1) Infant born at Parkland Health and Hospital Systems; 2) English or Spanish speaking mother; 3) Infant born at <31 weeks gestation; 4) Infant survival to 33 weeks post-menstrual age (PMA). Exclusion criteria: 1) Infant congenital anomalies: complex cardiac disease, neurological anomalies, suspected genetic condition, or ≥ two major body system congenital malformations; 2) Child Protective Services involvement resulting in supervision following discharge and/or displacement from biological parent; 3) Infant’s older sibling already enrolled within this study.

### Human rights, ethical considerations, informed consent; notice of IRB approval

The study was approved by the Institutional Review Board at UT Southwestern (STU 072018-095). ClinicalTrials.gov identifier: NCT03906435.

### Protocol

This was a randomized controlled trial (RCT) of five in-person CBT sessions versus routine care to assess the intervention impact on PPCV. Families were screened for eligibility and approached for enrollment at 33 weeks PMA; consented families were randomly assigned to either the intervention CBT group or the control treatment-as-usual group, utilizing block randomization of six. Multiple births were randomized to the same study group. A statistician generated the allocation sequence that was placed into a sealed envelope, opened, and assigned to each enrolled participant. The principal investigator screened, approached, enrolled, and assigned participants to their groups in accordance with the randomization allocation sequence envelopes. Due to the nature of the intervention, participants and intervention administers were not blinded. All parents were to complete questionnaires upon enrollment for baseline data at 33 weeks PMA and upon study completion at the child’s 6-month well-child check visit. All children received comprehensive primary care post-discharge in the NICU follow-up clinic, that included standard preventive medicine visits, anticipatory guidance, and social work support as needed. Adverse events to be followed were parental suicidality or infant death.

The control group received routine health care, anticipatory guidance, and health education throughout the NICU admission and post NICU discharge in the NICU follow-up clinic. The anticipatory guidance and health information were given by nursing, physicians, child life, social work, and subspecialty consulting services as applicable.

**Fig. 1:**
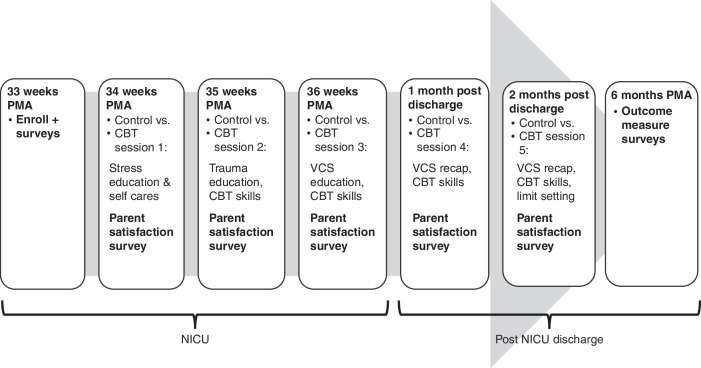
Methods timeline and cognitive behavioral therapy (CBT) intervention contents. CBT Cognitive behavioral therapy, VCS Vulnerable Child Syndrome, PMA post-menstrual age, VS. versus, NICU Neonatal Intensive Care Unit.

The intervention group received five manualized in-person CBT sessions focusing on concepts of NICU trauma and VCS in addition to routine health care provided to the control group. A written manual for the trauma-focused CBT sessions was created and adapted for this study to include PPCV and VCS content utilizing a previous effective study design.^17^ The sessions were created utilizing clinician focus groups to determine beneficial education for parents on stress and trauma symptom recognition, impacts of trauma on parental perceptions of the child; teach thought restructuring techniques to challenge trauma based reactions, and utilize skills to empower parents to recognize the most appropriate reaction to a current situation and thereby improve perceptions of child vulnerability, with practice scenarios created based upon common stressor events for NICU families at different time points in a child’s admission process and early life after discharge. This differed from previous CBT design in the fact that previous CBT manuals focused on trauma effects on parental mental health responses alone and not impacts on parental perceptions of their child as the child ages and leaves the NICU; previous CBT were also focused on delivering an intervention early in the NICU admission, not following discharge when stressors and mental health responses commonly re-emerge. CBT providers reviewed the treatment manual and were trained in the delivery of the intervention by two of the authors (MKH and RJS). Providers were given feedback during training to ensure all components of manual were delivered appropriately. Three providers completed training and administered the CBT manual to families; all CBT sessions were audio-recorded for fidelity rating.^18^ Parental satisfaction was measured via surveys given after each CBT session. See Fig. 1 for intervention timeline and contents.

### Measurements

#### 1. PPCV assessments

##### Vulnerable baby scale (VBSc)^15^

The VBSc is a 10-item questionnaire used to measure PPCV, given both at study enrollment and completion to assess the primary outcome of this study. Internal reliability is 0.7 within the VBSc, 0.7 with the VCSc.^15^ Test re-test reliability has not been documented. Vulnerable Child Scale (VCSc)^19^: The VCSc is an 8-item questionnaire used as a complementary secondary outcome measure of PPCV, given both at study enrollment and completion. Internal reliability is 0.74-0.75.^14,19^ Test-retest reliability is 0.96.^14^

##### Hoge-shaw revised vulnerable child scale (RVCSc)^20^

The RVCSc is a recently published 35-item questionnaire designed to measure PPCV severity and assess VCS diagnosis by incorporating child outcomes, given at study enrollment and completion. Scale incorporates sections of parental thoughts, feelings, and actions, and child outcomes. Likert scale of 0–4 with max score of 140. Used as an exploratory supplemental investigation of the concepts of PPCV and prediction of VCS development as an exploratory secondary outcome; validation studies are currently ongoing for this scale.

#### 2. Parent mental health assessments

##### Beck Anxiety Inventory (BAI)^21^

The BAI is a 21-item questionnaire used to measure parent anxiety scores and used as a secondary outcome for change over time throughout the study, given at study enrollment and completion. Test-retest reliability is 0.75 in the NICU parent population.^5^ Internal consistency in NICU parents is 0.9.^13^

##### Beck Depression Inventory—2nd Edition (BDI-II)^22^

The BDI-II is a 21-item questionnaire used to measure parent depression scores and used as a secondary outcome in change over time throughout the study, given at study enrollment and completion. Internal reliability is 0.92 in the NICU parent population.^5^ Test-retest reliability is 0.92.^13^

##### Traumatic Events Questionnaire (TEQ)^23^

The TEQ is an 11-item questionnaire that was used to assess specific past parent emotional traumatic experiences capable of eliciting post-traumatic stress symptoms as a secondary outcome, given at study enrollment and completion. Test-retest reliability for number of events is 0.91 and 0.72–1.0 for specific events.^13,23^

#### 3. Parent and children medical characteristics

Demographic Data on Parents and Baseline Medical Data on Infants: Data was collected on primary language, race, history of prior mental illness, insurance provider, and prior pregnancy histories. Data was collected on infant gestational age, Apgar scores, birth weight, and NICU diagnoses.

##### Statistical approach

Descriptive statistics were used to summarize variables at baseline and 6-9 months of age in the different groups. Chi-square statistical analysis was used to compare frequencies of primary and secondary outcomes between groups for categorical variables with a significance level (alpha) of 0.05. Two sample T-tests or Mann-Whitney U-tests were used to compare similar outcomes between groups for continuous variables depending upon distribution. SPSS (IBM, version 25) was used for all statistical analysis. Post-hoc analysis was conducted on VBSc variable using the Bayesian multinomial ordinal model with the MCMC procedure from SAS 9.4 (SAS Institute, Inc., Cary, NC).^24^

##### Sample size determination

Power analysis indicated that a total sample size of 54 with equal number of patients in each group would be required to have an 80% power for testing the primary hypothesis. This assumes that under the null hypothesis both the intervention and control group VBSc scores would decrease by 4 points with standard deviation equal to 2 from baseline to 6 months of age with no difference in baseline PPCV VBSc scores. The alternative hypothesis estimates the intervention group would decrease by 6 points with standard deviation equal to 3 from baseline to 6-9 months of age, thus decreasing 2 points more than the control group to detect a 50% effect size. The significance level (alpha) was set at 0.05 using a two sided two-sample t-test. These calculations were based on a previous study of VBSc scores that showed a 4-point decrease in VBSc scores in both the control and experimental group at 6 months compared with scores 4–5 weeks from baseline, with no significant difference between control and intervention.^13^

## Results

**Fig. 2:**
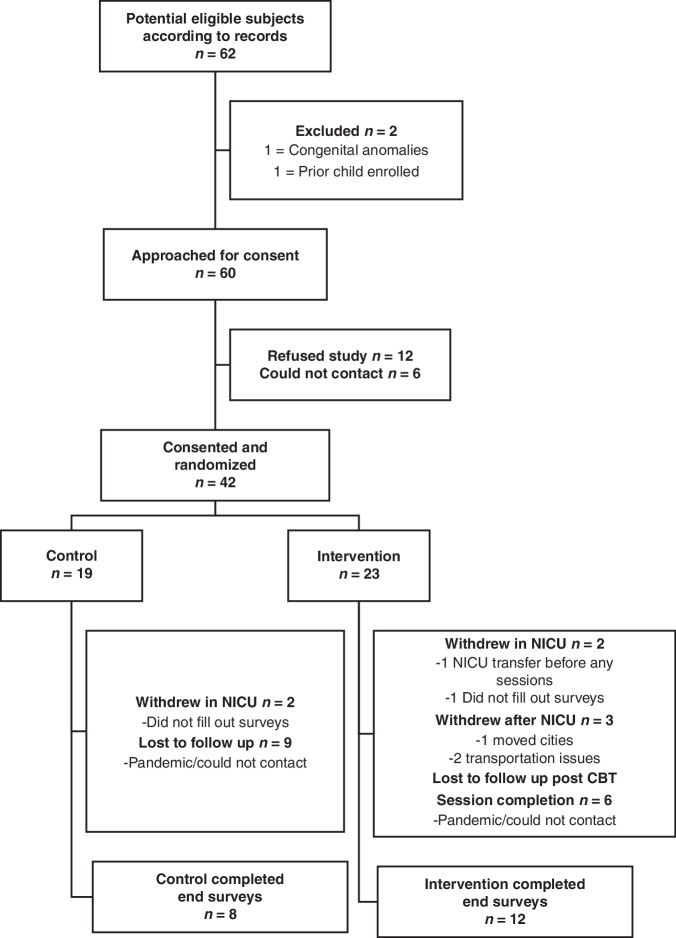
Study enrollment and completion rates flow diagram. NICU Neonatal Intensive Care Unit

Among 62 screened patients, 60 patients were eligible for the study, 2 met exclusion criteria, 12 families declined to participate, and 6 could not be reached despite three attempts. 42 families were randomized, and 20 families completed all components of the study protocol. Study enrollment was stopped early due to COVID-19 pandemic mandatory research pause by the institution, resulting in 77% of planned minimum enrolled sample size, and 37% of planned sample size that completed all study protocol. Details of the recruitment process are shown in Fig. 2. No crossover in groups occurred. Among the 42 families enrolled there were no significant differences in baseline maternal and infant demographics, PPCV, or parental mental health measures except for a slightly higher trauma score in the intervention group (Appendix Tables 5–7). Of the 8 control and 12 intervention families who completed the end point surveys, baseline maternal and infant demographics, PPCV, and parental mental health measures had no significant differences between the intervention and control groups (Tables 1–3). A sensitivity analysis showed that there was no difference in these baseline characteristics in those lost to follow up (Appendix Tables 8–10).

**Table 1:** Maternal Demographics, completed study.

	Control (*N* = 8)	Intervention (*N* = 12)
Maternal age in years mean ± SD	31 ± 6	29 ± 7
Single births, *N* (%)	6 (75)	8 (67)
Prior maternal mental health diagnosis, *N* (%)	0 (0)	1 (8)
Average Steroid Doses median (IQR)	1 (1, 1)	2 (1, 2)
C-Section Delivery, *N* (%)	7 (88)	7 (58)
English Primary Language, *N* (%)	3 (38)	8 (67)
Spanish Primary Language, *N* (%)	5 (62)	4 (33)
Hispanic Individuals, *N* (%)	6 (75)	9 (75)
African American Individuals, *N* (%)	2 (25)	3 (25)
White Individuals, *N* (%)	0 (0)	1 (5)
Medicaid, *N* (%)	8 (100)	12 (100)

None of the variables differed significantly between groups; *p*-value of <0.05 considered significant.

**Table 2:** Infant Demographics, completed study.

	Control (*N* = 8)	Intervention (*N* = 12)
Gestational Age, median (IQR)	29 (25, 30)	30 (28, 30)
Birthweight in grams, median (IQR)	985 (820, 1450)	1295 (1081, 1609)
PMA at discharge, median (IQR)	39 (39, 41)	38 (37, 41)
Sepsis, *N* (%)	4 (50)	4 (33)
Moderate/Severe BPD, *N* (%)	2 (25)	2 (17)
Retinopathy of Prematurity, *N* (%)	4 (50)	4 (33)
Intraventricular Hemorrhage, *N* (%)	0 (0)	0 (0)
Bassan Score, mode (range)	0 (0, 0)	0 (0, 0)
Necrotizing Enterocolitis, *N* (%)	1 (13)	1 (8)
Death, *N* (%)	0 (0)	0 (0)

*PMA* Post-menstrual age, *BPD* Bronchopulmonary Dysplasia.

None of the variables differed significantly between groups; *p*-value of <0.05 considered significant.

**Table 3:** Baseline parental PPCV and mental health outcomes, completed study.

	Control (*N* = 8)	Intervention (*N* = 12)
Baseline VBSc Score, median (IQR)	35 (25, 40)	33 (27, 36)
Baseline VCSc Score, median (IQR)	4 (0, 10)	6 (4, 9)
Baseline RVCSc Score, median (IQR)	55 (17, 57)	37 (20, 46)
Baseline BDI-II Score, median (IQR)	0 (0, 16)	10 (8, 17)
Baseline BAI Score, median (IQR)	8 (0, 15)	12 (4, 17)
Baseline TEQ Score, median (IQR)	1 (0, 1)	2 (0, 3)

*VBSc* Vulnerable Baby Scale, *VCSc* Vulnerable Child Scale, *RVCSc* Revised Vulnerable Child Scale, *BDI-II* Beck Depression Inventory 2nd Edition, *BAI* Beck Anxiety Inventory, *TEQ* Traumatic Event Questionnaire, *IQR* Interquartile Range.

None of the variables differed significantly between groups; *p*-value of <0.05 considered significant.

Primary outcome data analysis was performed for families that completed endpoint surveys. There was a 6-point median decrease in the VBSc score in the intervention group from the beginning of the study at 33 weeks PMA to the end of the study at 6 months chronological age. In contrast, measures demonstrated a zero-point median change in the VBSc score in the control group. The change in VBSc scores over time between groups neared significance with a *p*-value of 0.07 (See Table 4). Post-hoc analysis of VBSc score change over time for primary outcome was conducted using the Bayesian mutinomial ordinal model.^24^ Good convergence was obtained after merging patients into five groups. Bayesian analysis with a neutral prior indicated that the 5th centile of the posterior odds ratio (treatment vs control) was 1.25. Thus, there was 95% chance that the treatment group did better than the control.

**Table 4:** Primary and secondary outcomes of completed participants with end study data.

	Control group completed start and end surveys (*N* = 8)	Intervention group completed start and end surveys (*N* = 12)	*P*-value
Change in VBSc Score over time overall, median (IQR)	0.0 (−4.0, 0.75)	−6.0 (−8.5, −4.5)	0.07^**+**^
Change in VCSc Score over time overall, median (IQR)	0.0 (−4.75, 2.25)	−0.5 (−3.75, 3.75)	1.00
Change in RVCSc Score over time overall, median (IQR)	0.0 (−13.0, 6.0)	−17.0 (−22.0, 0.0)	0.27
Change in BDI-II Score over time overall, median (IQR)	0.0 (−5.0, 0.0)	−5.0 (−8.0, −0.5)	0.38
Change in BAI Score over time overall, median (IQR)	−4.0 (−9.0, 0.0)	−2.0 (−10.0, 5.25)	0.65
Change in TEQ over time overall, median (IQR)	−2.0 (−10.0, 5.25)	0.0 (−1.0, 0.0)	0.30
Parental Suicidality occurrences, *N* (%)	0 (0)	0 (0)	NA
Infant death, *N* (%)	0 (0)	0 (0)	NA

*VBSc* Vulnerable Baby Scale, *VCSc* Vulnerable Child Scale, *RVCSc* Revised Vulnerable Child Scale, *BDI-II* Beck Depression Inventory 2nd Edition, *BAI* Beck Anxiety Inventory, *TEQ* Traumatic Event Questionnaire, *IQR* Interquartile range.

None of the variables differed significantly between groups; *p*-value of <0.05 considered significant. + Post-hoc analysis of VBSc score change over time for primary outcome was conducted using the Bayesian multinomial ordinal model. Good convergence was obtained after merging patients into five groups. Bayesian analysis with a neutral prior indicated that the 5th centile of the posterior odds ratio (treatment vs control) was 1.25. Thus, there was 95% chance that the treatment group did better than the control.

For secondary outcomes, though VCSc and RVCSc scores did decrease over time in the intervention compared with no decrease in the control, there was no statistical difference in the change between the two groups. There were no statistically significant differences in parental depression, anxiety, or trauma scores from beginning of the study to the end of the study in either control or intervention group of those completing end-study surveys; there was a decrease in depression and anxiety scores over time in the intervention group, which was not seen in the control group (See Table 4). Fidelity and parent satisfaction ratings of the intervention. Fidelity ratings were 95–100% for all the CBT sessions, and parent satisfaction ratings for the CBT sessions were 4.9–5 out of 5, with 5 being the highest satisfaction.^18^ There was no positive suicidality ideation indicated in any parents; no enrolled infants died during the study period.

## Discussion

This single-center RCT evaluated the use of a novel manualized CBT intervention to reduce parental trauma and PPCV that are associated with the development of VCS.

Despite pandemic restrictions resulting in smaller than planned enrollment and primary outcome completion, decreased PPCV scores were observed in the intervention group favoring the trauma-informed CBT intervention over controls. This is the first intervention to our knowledge to demonstrate a reduction in PPCV scores. We speculate that a larger sample size may have shown a statistically significant difference.

By focusing our CBT intervention timeline surrounding NICU discharge, most traumatic NICU events have passed which allows parents to focus on learning CBT skills while still receiving NICU support, to anticipate stressful transition to home situations post-NICU discharge, and to allow for further practice of the skills once home with the additional coaching from a CBT provider in supplemental sessions post-NICU discharge. The CBT content also incorporated more education on trauma’s relation to effects of PPVC and VCS, teaching learnable and applicable skills to alter this trajectory utilizing CBT techniques. We believe that the intervention’s content focus on parenting issues including PPCV and VCS as well as the timing of CBT session administration surrounding NICU discharge likely contributed towards the success found in the study outcomes.

It was noted that in a previous study looking at CBT as an intervention for traumatic stress, depression, and anxiety, all domains decreased with intervention; the previous study occurred in the first 5 weeks of life when parental mental health symptoms are at their peak but gradually decline towards discharge, which is supported by the higher baseline BDI and BAI scores seen in this study compared to ours.^5^ Our intervention was designed to focus on trauma effects on PPCV and not directly anxiety and depression in contrast to the previous manual used to model our intervention. While our study was not powered to assess depression or anxiety effects, it looked at these measures as a secondary aim. Depression and anxiety did not decrease significantly in our intervention, however, there was still a slight decrease with our intervention over time. This could be because ratings were already lower at baseline or because of the CBT intervention’s content itself.

Strengths of this study include the randomized controlled design and the comparability of the study arms in baseline characteristics, except for a slightly higher trauma score in the intervention group, though not statistically significantly. That the baseline trauma score was higher in the intervention group and the trauma-informed CBT still had a positive impact supports the theory that trauma-based experiences in the NICU contribute towards NICU PPCV and the development of VCS.

Further strengths of the study include delivery of the intervention to English- and Spanish-speaking families with varying cultural backgrounds. There was no difference in the effectiveness of the intervention for these groups, which is important for generalizability. The population was also disproportionately insured by Medicaid, which is also another risk factor for progression of VCS, since this population often has fewer social support networks and more socioeconomic stressors.^12^ This study has implications for addressing health disparities in at-risk populations. The one barrier noted for this study population, which limited completion of the intervention post NICU discharge, was transportation. Otherwise, the intervention was feasible for an at-risk population.

The study started in April 2019 and was halted in March 2020 due to a mandatory study site research pause due to the emerging COVID-19 pandemic. This resulted in a 6-month study enrollment pause until the study team decided to end in–person subject participation due to confounding effects of COVID-19 pandemic stressors and research limitations on the outcomes. This decision resulted in a lower number of families enrolled than the calculated goal for power analysis. In the original power analysis, a change of 2 points or more decrease in the VBSc scores over the study period would have reached statistical significance with a total of 54 families. This would have been a 50% effect size when referencing the VBSc score natural progression data.^13^ Though not statistically significant, the 6-point VBSc score decrease in the CBT intervention group and a zero-point change in the control group signify that this novel intervention has promise to help parents reduce PPCV in their NICU graduates. This has the potential to reduce VCS in this population and therefore improve neurodevelopmental outcomes if the effect is sustained. This could be an important new treatment modality to consider in the NICU for improved longitudinal outcomes.

Study limitations include early termination of study due to the COVID-19 pandemic which also made it difficult to follow up with enrolled families, all of which resulted in a small sample size with primary outcome data despite many attempts to reach families. Of those lost to follow-up, there were no significant differences in the baseline maternal or infant characteristics nor in baseline mental health outcome measures. Therefore, loss to follow up should not have affected the results seen, however, this is still a possibility (see Appendix). Another limitation is that the study period occurred before the pandemic, where baseline stressors, anxiety, depression, trauma, and family dynamics were different. During and after the pandemic, mental health symptoms could be higher in incidence and severity. There could also be fewer modes of social support due to social distancing and family pandemic responses. In addition, results were based upon parent-report measures that are susceptible to bias. However, currently there are no other methods of assessing PPCV other than parent-report.

More research is needed to determine if post-pandemic factors impact the utility of the intervention. Further research is also needed to determine if other delivery methods of this novel CBT intervention such as telehealth might increase completed follow-up, reduce barriers to receiving therapy, and have the same impact as in-person participation. It will be important to determine if the intervention effect will extend beyond the study endpoint. It is possible that additional sessions may be needed to sustain effects. More research is also needed to determine the impact on child neurodevelopmental outcomes and VCS development. Parent-reported outcomes should be further compared against childhood neurodevelopmental and behavioral outcome variables to the potential impact on VCS in the population. Further research should also investigate the cost-effectiveness of the intervention for family-child outcomes and costs on the health care system utilization. There is the possibility that this intervention could prevent unnecessary “worried-well” visits in the health care system. There was anecdotal increased parenting confidence reported in the intervention group exhibited through qualitative interviews and testimonials, where parents utilized the CBT skills to appropriately manage their children’s symptoms at home without poor consequence to the child’s health. Parenting confidence and empowerment might also be objectively measured in future studies with parenting confidence scales.

## Conclusion

Our novel trauma-informed CBT intervention demonstrated a reduction in PPCV, which may decrease the risk of VCS development in an at-risk population of preterm infant’s parents. To our knowledge, this is the first published intervention to demonstrate these findings. Although results need to be replicated in a larger study to reach expected sample size for power analysis, our findings show a strong signal favoring intervention. Further research is needed to correlate the PPCV reduction to any difference in their children’s neurodevelopmental outcomes seen in VCS. This could prove a valuable supplemental mode of therapy to improve NICU children’s long-term neurodevelopmental outcomes and parent-infant outcomes.

## Supplementary information

checklist

## Data Availability

ClinicalTrials.gov organizes information for each registered study as an integrated unit, displaying the study protocol information and, if available, the corresponding results information on the same page under different tabs.

## Appendix

Table 5

**Table 5:** Maternal Demographics, all enrolled.

	Control (*N* = 19)	Intervention (*N* = 23)
Maternal age in years mean ± SD	30 ± 5	29 ± 7
Single births, *N* (%)	15 (79)	19 (83)
Prior maternal mental health diagnosis, *N* (%)	1 (5)	1 (4)
Average Steroid Doses, median (IQR)	1 (1, 2)	1 (1, 2)
C-Section Delivery, *N* (%)	14 (74)	14 (61)
English Primary Language, *N* (%)	7 (37)	15 (65)
Spanish Primary Language, *N* (%)	12 (63)	8 (36)
Hispanic Individuals, *N* (%)	14 (74)	18 (78)
African American Individuals, *N* (%)	5 (26)	4 (17)
White Individuals, *N* (%)	0 (0)	1 (4)
Medicaid, *N* (%)	19 (100)	22 (95)

None of the variables differed significantly between groups; *p*-value of <0.05 considered significant.

Table 6

**Table 6:** Infant Demographics, all enrolled.

	Control (*N* = 19)	Intervention (*N* = 23)
Gestational Age in weeks, median (IQR)	28 (26, 30)	29 (26, 30)
Birthweight in grams, median (IQR)	1020 (850, 1550)	1190 (995, 1510)
PMA at discharge, median (IQR)	40 (38, 44)	39 (37, 42)
Sepsis, *N* (%)	7 (37)	10 (44)
Moderate/Severe BPD, *N* (%)	4 (21)	5 (22)
Retinopathy of Prematurity, *N* (%)	9 (47)	10 (43)
Intraventricular Hemorrhage, *N* (%)	0 (0)	2 (9)
Bassan Score, mode (range)	0 (0, 1)	0 (0, 0)
Necrotizing Enterocolitis, *N* (%)	1 (5)	2 (9)
Death, *N* (%)	0 (0)	0 (0)

*PMA* Post-menstrual age, *BPD* Bronchopulmonary Dysplasia.

None of the variables differed significantly between groups; *p*-value of <0.05 considered significant.

Table 7

**Table 7:** Baseline Survey Scores comparing intervention with control at enrollment for those that have baseline survey data, all enrolled: intervention (*N* = 17) vs. control (*N* = 18)

	Control (*N* = 17)	Intervention (*N* = 18)	*P*-value
Baseline VBSc Score, median (IQR)	36 (30, 40)	33 (28, 37)	0.71
Baseline VCSc Score, median (IQR)	6 (3, 10)	8 (5, 10)	0.44
Baseline RVCSc Score, median (IQR)	51 (33, 56)	37 (20, 45)	0.11
Baseline BDI-II Score, median (IQR)	2 (0, 19)	9 (4, 17)	0.34
Baseline BAI Score, median (IQR)	4 (0, 15)	10 (3, 17)	0.46
Baseline TEQ, median (IQR)*	0 (0, 2)	1 (1, 3)	0.03

*VBSc* Vulnerable Baby Scale, *VCSc* Vulnerable Child Scale, *RVCSc* Revised Vulnerable Child Scale, *BDI-II* Beck Depression Inventory 2nd Edition, *BAI* Beck Anxiety Inventory, *TEQ* Traumatic Event Questionnaire, *IQR* Interquartile Range.

Differed significantly with a p-value of 0.03. All other variables did not differ significantly between groups.

Table 8

**Table 8:** Maternal Demographics.

	VBSc at 6–9 months (*N* = 20)	Do not have VBSc at 6–9 months (*N* = 22)
Intervention	12 (60)	11 (50)
Control	8 (40)	11 (50)
Maternal age in years mean ± SD	30 ± 7	29 ± 6
Single births, *N* (%)	14 (70)	20 (91)
Prior maternal mental health diagnosis, *N* (%)	1 (5)	1 (5)
Average Steroid Doses, median (IQR)	1 (1, 2)	1 (1, 2)
C-Section Delivery, *N* (%)	14 (70)	14 (64)
English Primary Language, *N* (%)	11 (55)	11 (50)
Spanish Primary Language, *N* (%)	9 (45)	11 (50)
Hispanic Individuals, *N* (%)	15 (75)	17 (77)
African American Individuals, *N* (%)	5 (25)	4 (18)
White Individuals, *N* (%)	0 (0)	1 (5)
Medicaid, *N* (%)	20 (100)	21 (95)

Comparing those that have primary outcome (VBSc) at 6–9 months (*N* = 20) compared to those that do not *(N* = 22).

*VBSc* Vulnerable Baby Scale.

None of the variables differed significantly between groups; *p*-value of <0.05 considered significant.

Table 9

**Table 9:** Infant Demographics. Comparing those that have primary outcome (VBSc) at 6–9 months (*N* = 20) compared to those that do not (*N* = 22)

	VBSc at 6–9 months (*N* = 20)	Do not have VBSc at 6–9 months (*N* = 22)
GA median (IQR)	29 (27, 30)	27 (25, 30)
Birthweight in grams, median (IQR)	1170 (969, 1569)	1063 (918, 1413)
PMA in weeks at discharge, median (IQR)	39 (38, 41)	41 (37, 43)
Sepsis, *N* (%)	8 (40)	9 (41)
Moderate/Severe BPD, *N* (%)	4 (20)	5 (23)
ROP, *N* (%)	8 (40)	11 (50)
IVH, *N* (%)	0 (0)	2 (9)
Bassan Score, mode (range)	0 (0, 0)	0 (0, 1)
NEC, *N* (%)	2 (10)	1 (5)
Death, *N* (%)	0 (0)	0 (0)

*VBSc* Vulnerable Baby Scale, *GA* Gestational age, *IQR* Interquartile range, *PMA* Post menstrual age, *BPD* Bronchopulmonary dysplasia, *ROP* retinopathy of prematurity, *IVH* intraventricular hemorrhage, *NEC* necrotizing enterocolitis.

None of the variables differed significantly between groups; *p*-value of <0.05 considered significant.

Table 10

**Table 10:** Baseline Survey Scores comparing those that have primary outcome (VBSc) at 6–9 months (*N* = 20) compared to those that do not (*N* = 15) for those who have baseline survey data

	VBSc at 6–9 months (*N* = 20)	Do not have VBSc at 6–9 month (*N* = 15)
Baseline VBSc Score, median (IQR)	33 (27, 40)	34 (31, 39)
Baseline VCSc Score, median (IQR)	6 (2, 9)	8 (5, 12)
Baseline RVCSc Score, median (IQR)	37 (22, 55)	49 (35, 53)
Baseline BDI-II Score, median (IQR)	9 (1, 17)	7 (1, 17)
Baseline BAI Score, median (IQR)	11 (4, 16)	4 (0, 17)
Baseline TEQ Score, median (IQR)	1 (0, 3)	1 (0, 2)

None of the variables differed significantly between groups; *p*-value of <0.05 considered significant.

*VBSc* Vulnerable Baby Scale, *VCSc* Vulnerable Child Scale, *RVCSc* Revised Vulnerable Child Scale, *BDI-II* Beck Depression Inventory 2nd Edition, *BAI* Beck Anxiety Inventory, *TEQ* Traumatic Event Questionnaire, *IQR* Interquartile Range
